# Dementia’s mortality in America: a local, regional, and temporal evaluation

**DOI:** 10.1590/1980-5764-DN-2025-0298

**Published:** 2026-02-06

**Authors:** Lucas Casagrande Passoni Lopes

**Affiliations:** 1 Universidade de São Paulo, Faculdade de Medicina de Bauru, Bauru SP, Brazil.

**Keywords:** Dementia, Americas, Mortality, Demência, América, Mortalidade

## Abstract

Dementia is a leading global health challenge. Despite its burden, data from American countries remain scarce and fragmented. This lack of robust evidence impedes regional planning and policy-making.

**Objective:**

The aim of the study was to evaluate the temporal trends of dementia’s mortality in America with a local and regional approach.

**Methods:**

This ecological time-series study analyzed age-adjusted dementia mortality rates across American countries between 2000 and 2019. Data were obtained from Pan American Health Organization and cross-validated with national and international sources. Joinpoint regression was used to estimate annual percent change, identifying significant temporal trends in mortality.

**Results:**

Between 2000 and 2019, dementia mortality rates were highest in the Insular Americas and varied widely across countries. The United States, Canada, and several Central and South American nations exhibited elevated rates, while others, such as Paraguay and Venezuela, reported consistently low values. National trends were heterogeneous, with some countries showing steady declines and others persistent or erratic increases.

**Conclusion:**

This study highlights significant temporal trends in American dementia mortality, identifying critical areas for public health intervention and offering a foundation for future research on healthcare equity and cancer outcomes.

## RESUMO.

A demência é um dos principais desafios globais para a saúde. Apesar de sua relevância, os dados dos países americanos permanecem escassos e fragmentados. Essa falta de evidências robustas impede o planejamento regional e a formulação de políticas.

**Objetivo:**

Avaliar as tendências temporais da mortalidade por demência nas Américas com uma abordagem local e regional.

**Métodos:**

Este estudo ecológico de séries temporais analisou as taxas de mortalidade por demência ajustadas por idade nos países americanos entre 2000 e 2019. Os dados foram obtidos da Organização Pan-Americana da Saúde e submetidos à validação cruzada com fontes nacionais e internacionais. A regressão por pontos de junção foi utilizada para estimar a variação percentual anual, identificando tendências temporais significativas na mortalidade.

**Resultados:**

Entre 2000 e 2019, as taxas de mortalidade por demência foram mais altas nas Américas insulares e variaram amplamente entre os países. Os Estados Unidos, Canadá e vários países da América Central e do Sul apresentaram taxas elevadas, enquanto outros, como Paraguai e Venezuela, relataram valores consistentemente baixos. As tendências nacionais foram heterogêneas, com alguns países apresentando declínios constantes e outros aumentos persistentes ou erráticos.

**Conclusão:**

Este estudo destacou tendências temporais significativas na mortalidade por demência nos Estados Unidos, identificando áreas críticas para intervenção em saúde pública e oferecendo uma base para pesquisas futuras sobre equidade em assistência médica e resultados do câncer.

## INTRODUCTION

 Dementia is a clinical syndrome marked by a progressive decline in cognitive functions that interferes with an individual’s ability to perform everyday activities^1^ . It results from a range of underlying pathologies that cause neuronal damage and brain atrophy. Although more common in older adults, dementia can affect individuals across age groups depending on the etiology^1^ . 

 Alzheimer’s disease is the most prevalent cause of dementia, followed by vascular dementia, Lewy body dementia, and frontotemporal dementia^2^ . While memory impairment is a hallmark symptom, especially in Alzheimer’s, dementia also affects language, executive function, attention, and behavior, progressively leading to loss of autonomy^2^ . 

 Dementia represents a growing global public health crisis. As of 2023, over 55 million people live with dementia worldwide, and this number is projected to exceed 150 million by 2050, largely driven by aging populations^3^ . The associated economic burden is staggering, surpassing US$ 1.3 trillion annually, including healthcare costs and unpaid caregiving. Beyond financial strain, dementia significantly impacts families and caregivers, contributing to emotional distress and reduced quality of life^3^ . 

 Despite its global relevance, the understanding and monitoring of dementia in the American continent remain limited^4^ . This is concerning, as countries in the region face rapidly aging populations, limited healthcare infrastructure, and socioeconomic disparities that may heighten vulnerability to dementia^4^ . The lack of consistent epidemiological data hinders the formulation of effective public policies and health planning^4^ . 

 Organizations such as the Pan American Health Organization (PAHO) have sought to map the dementia landscape in the region. However, raw data alone are insufficient^4^ . A deeper analysis and contextual refinement of these data are crucial to make them accessible, actionable, and aligned with local realities^4^ . Enhancing the quality and applicability of information can democratize access to evidence and guide strategic decisions^3,4^. In this context, the present study aims to evaluate the temporal trends of dementia’s mortality across the American continent, providing insights about the local and regional differences. 

## METHODS

### Study design

 This ecological study aimed to evaluate the temporal trends of dementia’s mortality in the American continent between 2000 and 2019, highlighting local and regional disparities. 

### Data source

 Mortality data were primarily obtained from the PAHO Mortality Information System, accessed via https://www.paho.org/en. More specifically, data were retrieved from the reports section periodically published by PAHO. PAHO is specialized in health promotion, disease prevention, and epidemiological analysis, among other actions in the aforementioned location. To enhance data validity, they were cross-referenced with complementary sources, including national vital statistics offices, the World Health Organization (WHO) Global Health Estimates, the Global Burden of Disease Study (GBD), and databases from the United Nations Economic Commission for Latin America and the Caribbean. 

### Inclusion Criteria

 Only countries within the Americas that reported complete annual data on dementia-related mortality (International Code of Diseases 9th edition — ICD-9 — 290 to 294, and ICD-10 codes F01, F03, and G30) during the period 2000–2019 were included in the analysis. Mortality rates were age-adjusted per 100,000 population. 

### Data extraction and processing

 Data were manually extracted from PAHO’s platform in January 2025 and organized into Excel® spreadsheets. Each dataset included variables such as country, year, number of dementia-related deaths, and age-standardized mortality rate. Countries lacking consistent or complete mortality data for at least 10 consecutive years within the study period were excluded. 

### Data analysis

 The data were interpreted using two software programs: R 4.4.3® and Joinpoint 5.4.0®. In this case, using Joinpoint, it was possible to evaluate the temporal trends of the data through the interpretation of the Annual Percentage Change (APC). This parameter indicates whether there are increasing (APC > 0 and p-value ≤ 0.05), decreasing (APC < 0 and p-value ≤ 0.05), or stable (p-value > 0.05) trends in dementia mortality in countries of the Americas. The following formula summarizes the concept of the performed analysis. The R software, in turn, assisted in the production of some of the figures that illustrate the results obtained through the aforementioned analysis. ln(yt) = 0 + j=1k + 1j ( t - j - 1) + t Where y represents dementia-related mortality rates; t, the time (2000 - 2019); ln(yt), the natural logarithm of the mortality rates value, used to linearize the trend; 0, the intercept, corresponding to the estimated baseline level at the beginning of the study period; j, the slope coefficients, each representing the rate of change within a specific time segment; j , the joinpoints, that is, the points in time where a significant change in trend occurs; k, the number of joinpoints identified in the analysis; and, the random error term. 

## RESULTS

**Figure 1:**
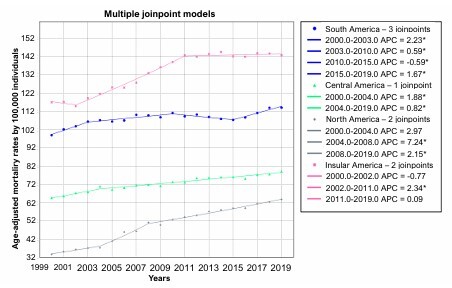
Temporal trends in age-adjusted mortality rates by dementia across the American continent regions between 2000 and 2019.

 From a regional standpoint, dementia mortality rates were consistently highest in the Insular Americas, followed by South America, Central America, and North America throughout the entire study period. The Insular Americas experienced a marked increase in mortality between 2002 and 2011, after which rates stabilized. In South America, mortality rose from 2000 to 2010, declined slightly until 2015, and then resumed an upward trajectory. Central America showed a sustained increase in mortality throughout the period, although the pace of growth varied over time. North America displayed stable rates from 2000 to 2004, followed by a steady increase thereafter (Figure 1). 

**Figure 2:**
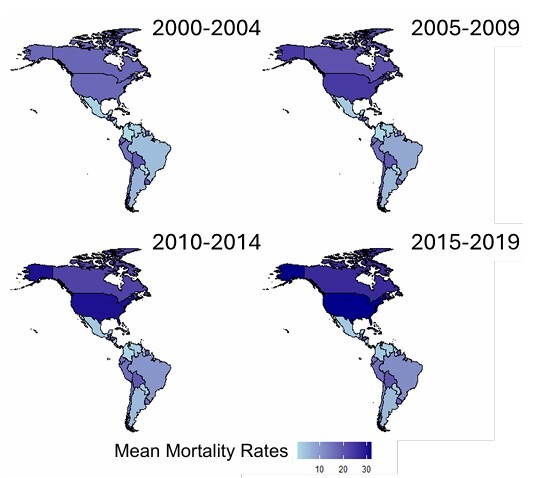
Heatmap representing the age-adjusted mortality trends by dementia across the American continent between 2000 and 2019.

 Among individual countries, the United States and Canada exhibited the highest mortality rates during the observed period. Notably, Honduras and Nicaragua also recorded disproportionately high mortality rates, as did Bolivia, Peru, Ecuador, and Uruguay. In contrast, Paraguay, Mexico, Venezuela, and Colombia consistently reported the lowest mortality rates (Figure 2). 

 Argentina experienced a steady and continuous decline in dementia mortality. Brazil underwent a sharp increase from 2000 to 2004, followed by slower but persistent growth until 2019. Paraguay followed a similar trajectory, with an early surge that later stabilized. Conversely, Ecuador and Uruguay both displayed significant and sustained reductions in mortality over time. Chile’s rates declined substantially after 2009, whereas Colombia exhibited irregular fluctuations without a consistent trend. Suriname showed a complex pattern marked by an initial decrease, followed by an increase, and eventual stabilization. Venezuela’s mortality trends were highly erratic, alternating between periods of rise and decline. 

 In North America, the United States demonstrated a marked increase in dementia mortality until 2010, which was followed by a slower, yet still significant, rise. Mexico’s rates increased until approximately 2009, then plateaued. Canada’s trajectory was mixed, with a sharp rise from 2004 to 2008, a subsequent period of stability, and another substantial increase beginning in 2011. 

 In Central America, Belize exhibited a triphasic trend: a modest increase until 2010, a decline until 2016, and a renewed rise thereafter. Costa Rica recorded a significant increase in early years, followed by a stable phase and another surge after 2016. El Salvador experienced a gradual decline over the full period. Guatemala showed an early rise, then stability, and a later decrease. Honduras displayed alternating trends, with early increases and decreases, followed by a pronounced spike between 2010 and 2013, and stabilization afterward. Nicaragua experienced an extraordinary increase between 2000 and 2002, followed by a prolonged stable trend. Panama remained mostly stable, with a brief but substantial rise between 2012 and 2015. 

 In the Insular Americas, Antigua and Barbuda showed extreme fluctuations — an initial sharp decline, followed by a dramatic rise until 2007, and moderate increases thereafter. The Bahamas saw increasing mortality until 2008 and a gradual decline afterward. Barbados experienced a marked decline early on, followed by strong increases, particularly between 2007 and 2010, and eventual stabilization. Cuba’s mortality steadily increased until 2009, then remained unchanged. Grenada’s rates dropped until 2008, surged sharply through 2011, and then stabilized. Trinidad and Tobago exhibited a significant decline from 2010 to 2019. The Dominican Republic remained mostly stable, with only a brief period of increase. Jamaica and Saint Lucia showed no significant trends throughout the study period. Haiti’s mortality remained largely unchanged as well. 

Supplementary Material 1 (Figures) — available at  https://www.demneuropsy.org/wp-content/uploads/2025/09/DN-2025.0298-Supplementary-Material-1.docx and Supplementary Material 2 (Tables) — available at  https://www.demneuropsy.org/wp-content/uploads/2025/09/DN-2025.0298-Supplementary-Material-2.xlsx present the trends of each evaluated country. 

## DISCUSSION

 Dementia mortality trends varied considerably across the Americas from 2000 to 2019. The Insular Americas led consistently with the highest mortality rates, followed by South, Central, and North America. Country-level analysis revealed elevated mortality in the United States and Canada, and unexpectedly high levels in parts of Central and South America, including Honduras, Nicaragua, Bolivia, Peru, and Ecuador. Meanwhile, countries such as Paraguay, Venezuela, Mexico, and Colombia maintained relatively low mortality rates. Temporal patterns also varied: some countries, like Argentina, Ecuador, and Uruguay, experienced steady declines, while others, such as Brazil, Paraguay, and the United States, showed persistent increases. Several nations, particularly in the Caribbean, exhibited complex or erratic trajectories, reflecting regional heterogeneity in dementia mortality patterns. 

 Dementia-related mortality rates were consistently highest in the Insular Americas, followed by South, Central, and North America, reflecting a broad gradient of social, economic, and healthcare disparities^5 ,6^. The Caribbean region, despite its relatively smaller population size, has faced disproportionate challenges such as underfunded health systems, limited geriatric care infrastructure, and high rates of comorbidities like hypertension and diabetes, which contribute significantly to dementia risk and mortality^5^ . In contrast, countries like the United States and Canada, despite having more robust healthcare systems, still reported elevated mortality — particularly among marginalized groups — highlighting the persistent influence of social determinants such as race, income inequality, and regional disparities in healthcare access^6^ . Moreover, the unexpectedly high dementia mortality rates observed in parts of Central and South America — including Honduras, Nicaragua, Bolivia, Peru, and Ecuador — may stem from a combination of delayed diagnosis, cultural stigma surrounding cognitive decline, low investment in elder care, and inadequate tracking of neurodegenerative conditions in national surveillance systems^6^ . 

 Conversely, countries such as Paraguay, Venezuela, Mexico, and Colombia maintained relatively low dementia-related mortality rates throughout the study period. While lower reported mortality could partly reflect genuine protective factors — such as stronger family-based caregiving traditions or dietary and lifestyle patterns — underreporting and limited diagnostic capacity may also obscure the true burden of disease^7^ . In some cases, competing mortality from infectious diseases or violence may reduce the number of individuals surviving to ages when dementia typically manifests^7^ . Additionally, differences in the classification and coding of dementia-related deaths in death certificates may contribute to the observed variability, underscoring the role of national health information systems in shaping mortality statistics^8^ . 

 Temporal trends revealed important divergences across countries. Nations such as Argentina, Ecuador, and Uruguay experienced steady declines in dementia mortality, possibly due to improvements in healthcare access, early detection policies, and better management of cardiovascular and metabolic comorbidities^9^ . Public health efforts in these countries may have also benefited from long-standing investments in education and social protection systems, which are known to be protective against cognitive decline^9,10^. Furthermore, increased awareness and destigmatization of dementia may have facilitated earlier intervention and better care, potentially improving survival and quality of life for affected individuals^8,10^ . 

 In contrast, countries like Brazil, Paraguay, and the United States showed persistent increases in dementia-related mortality over time. In Brazil, rapid population aging outpaced the development of age-appropriate health services, while high levels of inequality, regional disparities in healthcare infrastructure, and underdiagnosis in rural or underserved areas may have fueled the upward trend^6,11^. The United States also experienced a notable increase, particularly in southern states and among racial minorities, suggesting that structural inequities in healthcare access, educational attainment, and the management of chronic conditions remain powerful drivers of dementia mortality^12^ . In Paraguay, the persistent rise may reflect the dual burden of demographic transition and insufficient health system preparedness for neurodegenerative diseases^8,13^ . 

 Several countries in the Insular Americas exhibited highly variable or erratic mortality trajectories, revealing the complexity and heterogeneity of dementia epidemiology in the region^14,15^. These fluctuations may be due to inconsistencies in health surveillance systems, political and economic instability affecting healthcare delivery, and changes in international aid or national health priorities^14^ . Moreover, periodic natural disasters and migration waves have disrupted care continuity for older adults in these areas, further complicating mortality patterns. The observed heterogeneity underscores the need for context-sensitive approaches that account for local vulnerabilities and health system capacities^15^ . 

 These findings have direct implications for public health planning and policy^16-18^. Efforts to reduce dementia-related mortality must go beyond clinical care and address broader social determinants of health, including education, income, gender inequality, and regional disparities in healthcare infrastructure^16^ . Policies that promote early detection, community-based care models, and caregiver support programs can mitigate the impact of dementia, particularly in resource-constrained settings^17^ . Moreover, integrating dementia prevention into non-communicable disease control strategies — by addressing hypertension, diabetes, physical inactivity, and poor nutrition — can yield long-term benefits^17^ . Ultimately, a regionally coordinated approach, tailored to the demographic and cultural realities of each country, will be essential to reduce the burden of dementia and improve outcomes for aging populations across the Americas^18^ . 

 This study leverages standardized, internationally recognized mortality data over a two-decade span, providing a comprehensive view of dementia trends in the Americas. The use of joinpoint regression enhances the precision in detecting meaningful changes in mortality trajectories. Additionally, data triangulation from multiple sources (PAHO, WHO, GBD, and national databases) increases the robustness and reliability of findings, offering actionable insights for public health decision-makers. 

 The study is limited by the ecological design, which precludes individual-level inferences. Variations in the quality, completeness, and certification practices of mortality data across countries may introduce bias or underreporting. Furthermore, the exclusion of countries with incomplete datasets may limit the generalizability of findings to the entire region. Despite cross-validation efforts, disparities in diagnostic accuracy and death certification for dementia remain significant challenges. 

 In conclusion, this study provides a comprehensive analysis of dementia’s mortality trends in the Americas from 2000 to 2019, highlighting significant variations in disease dynamics over time. The findings underscore the necessity of continuous surveillance, improved reporting accuracy, and targeted interventions to curb the increasing burden of dementias in the region. Given the observed trends, future research should focus on identifying specific drivers of incidence changes, including socioeconomic, health care access, and structural factors. Strengthening international collaborations and optimizing control measures will be essential to mitigate the impact of dementias and enhance public health preparedness for their growing demand. 

## Data Availability

Data derived from public domain resources. The data that support the findings of this study are available in https://www.paho.org/en.
