# Supplementary material for: Dementia’s mortality in America: a local, regional, and temporal evaluation
Source: Dement Neuropsychol. 2026 Feb 6;20:e20250298. doi: 10.1590/1980-5764-DN-2025-0298 (PMC12885473; doi:10.1590/1980-5764-DN-2025-0298)
Supplement: Supplementary Material 1 [file 1980-5764-dn-20-e20250298-md1.docx]

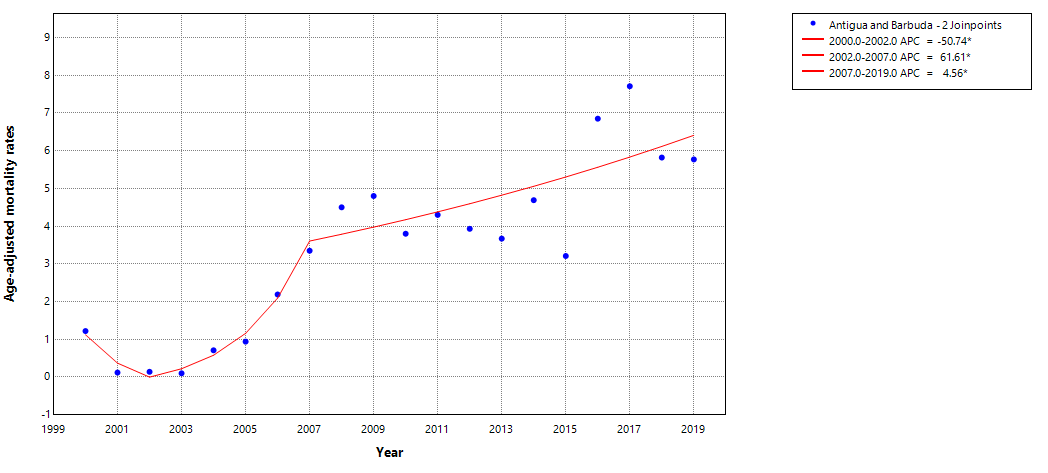
Supplementary figure 1 - Temporal trends in age-adjusted mortality by dementia in Antigua and Barbuda between 2000 and 2019


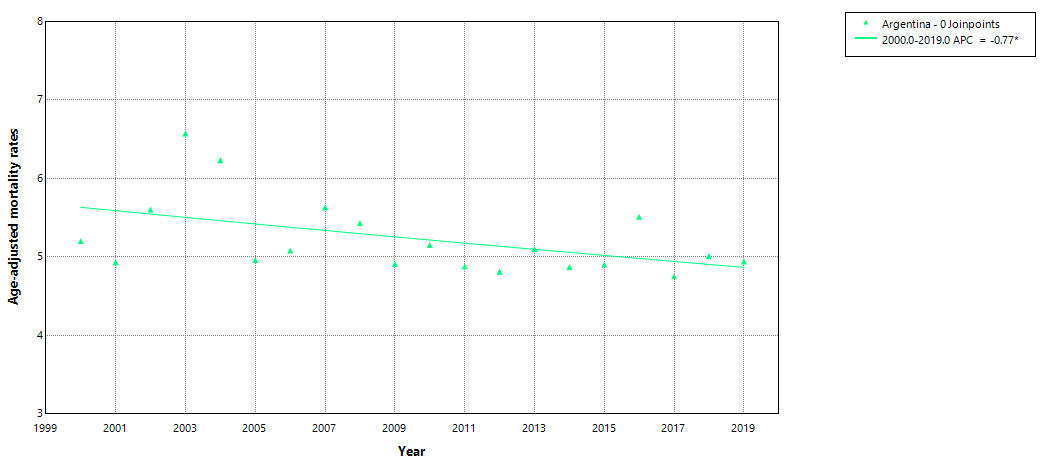


Supplementary figure 2 - Temporal trends in age-adjusted mortality by dementia in Argentina between 2000 and 2019


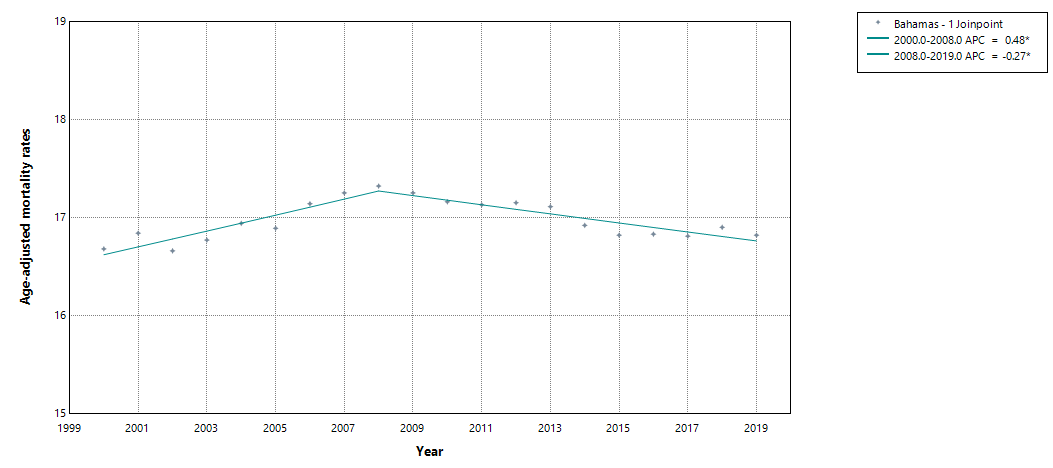


Supplementary figure 3 - Temporal trends in age-adjusted mortality by dementia in Bahamas between 2000 and 2019


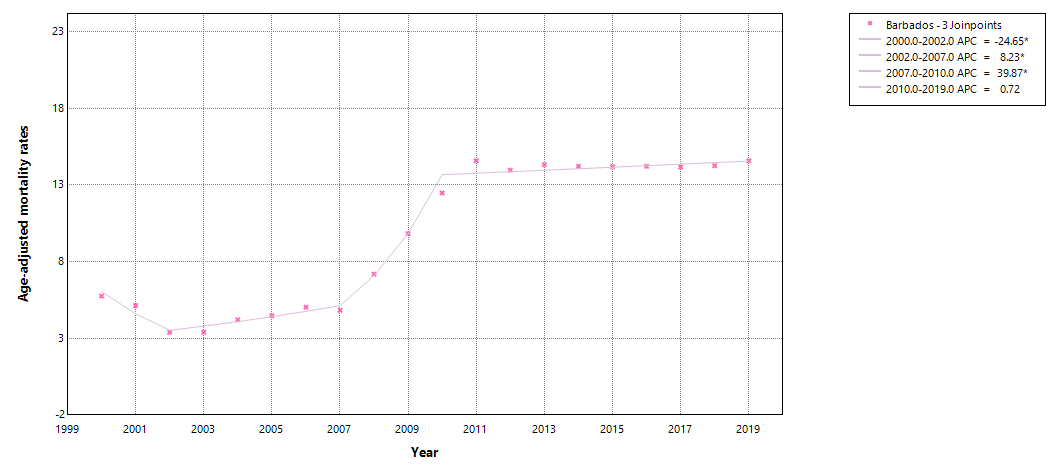


Supplementary figure 4 - Temporal trends in age-adjusted mortality by dementia in Barbados between 2000 and 2019


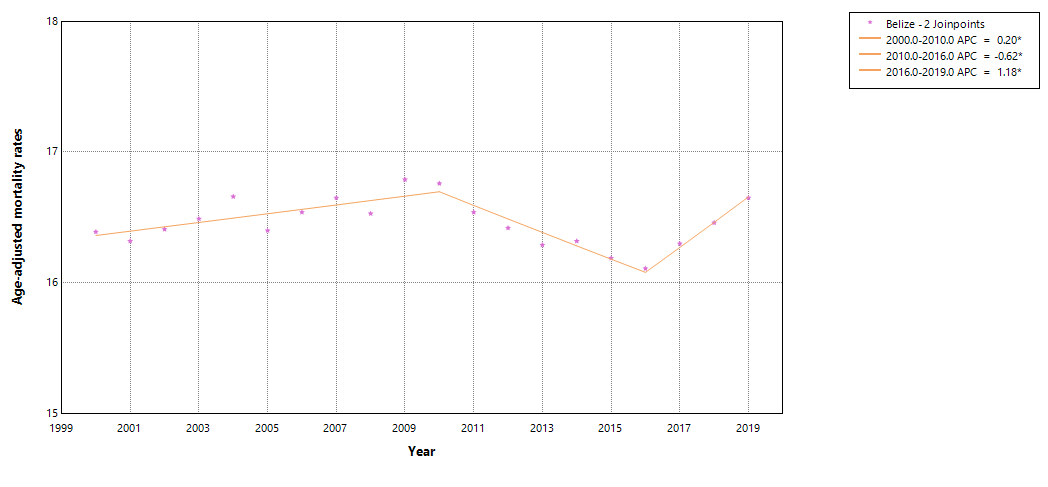


Supplementary figure 5 - Temporal trends in age-adjusted mortality by dementia in Belize between 2000 and 2019


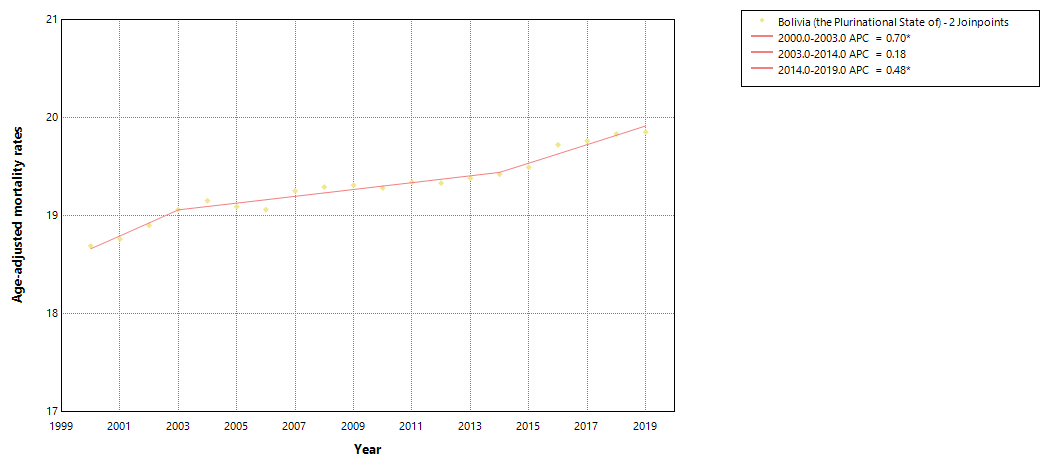


Supplementary figure 6 - Temporal trends in age-adjusted mortality by dementia in Bolivia between 2000 and 2019


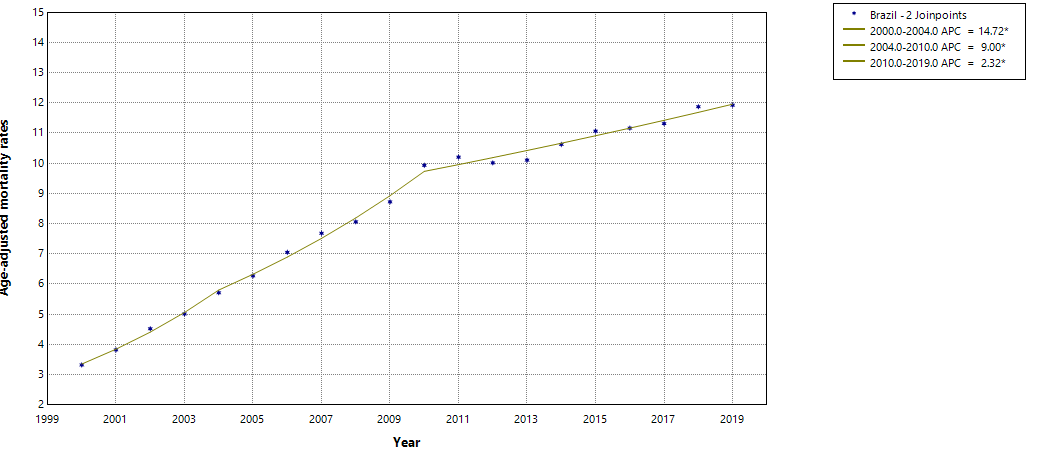


Supplementary figure 7 - Temporal trends in age-adjusted mortality by dementia in Brazil between 2000 and 2019


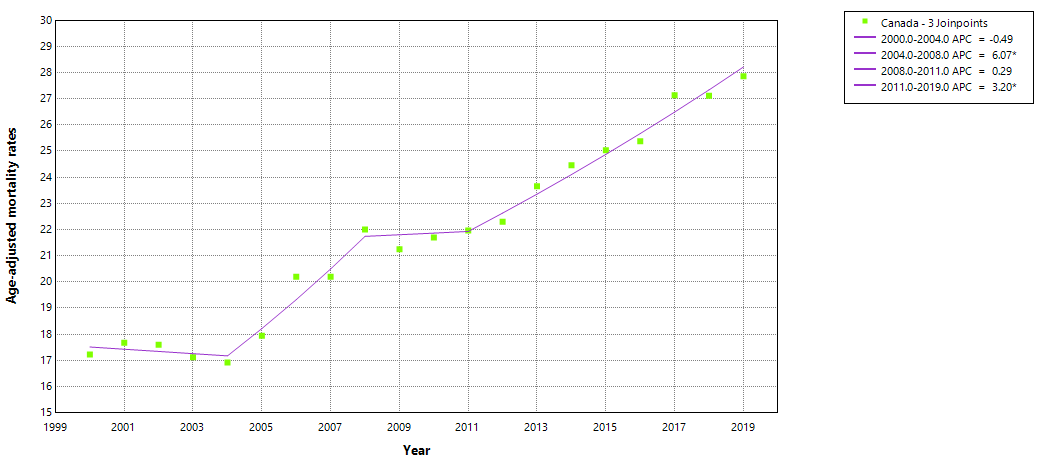


Supplementary figure 8 - Temporal trends in age-adjusted mortality by dementia in Canada between 2000 and 2019


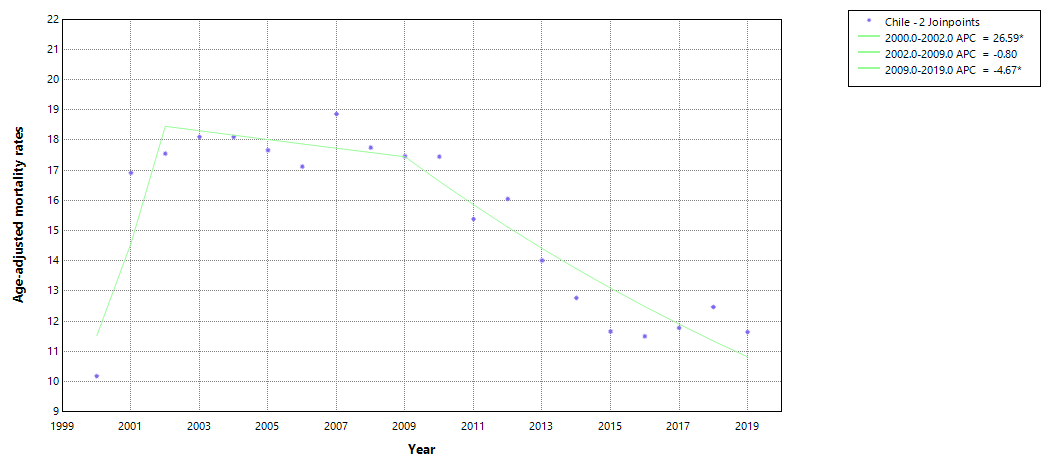


Supplementary figure 9 - Temporal trends in age-adjusted mortality by dementia in Chile between 2000 and 2019


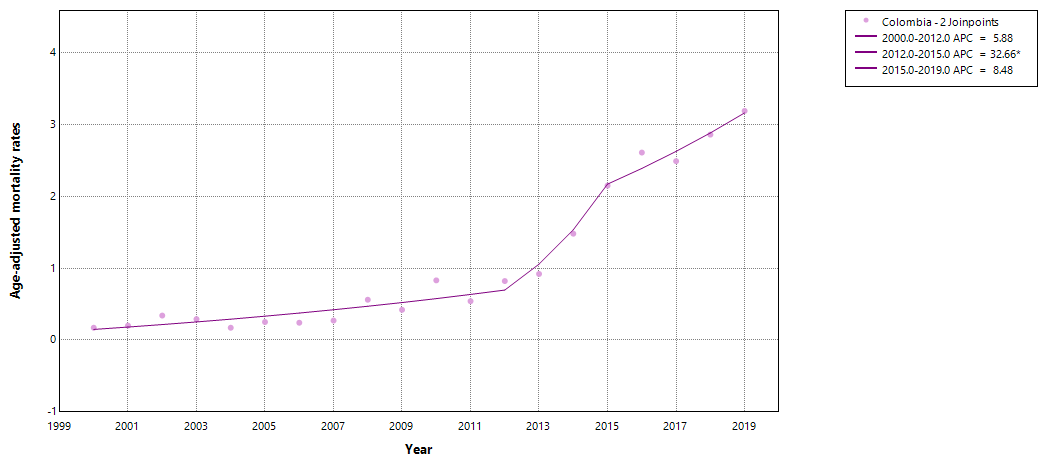


Supplementary figure 10 - Temporal trends in age-adjusted mortality by dementia in Colombia between 2000 and 2019


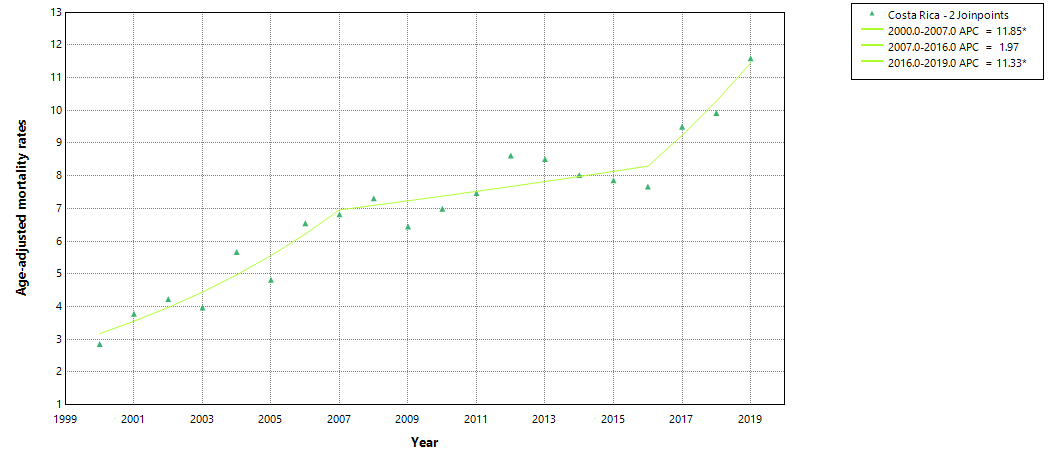


Supplementary figure 11 - Temporal trends in age-adjusted mortality by dementia in Costa Rica between 2000 and 2019


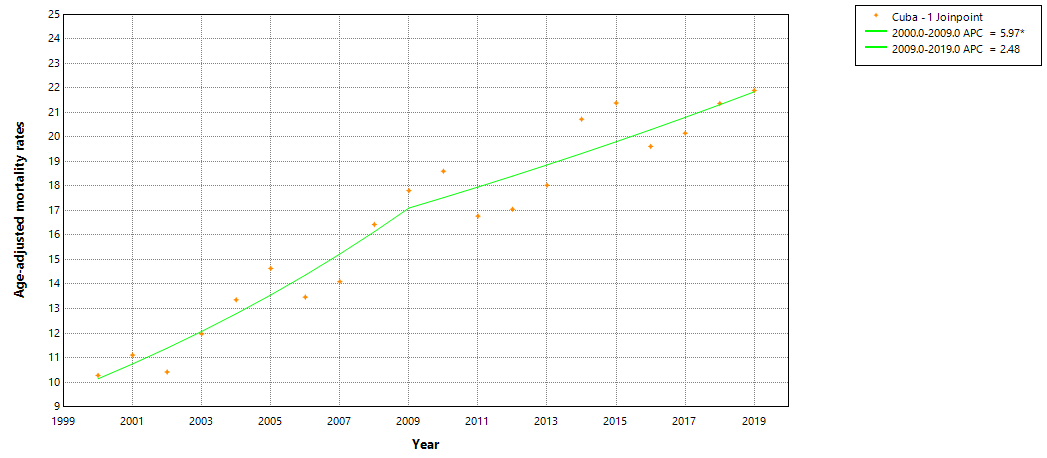


Supplementary figure 12 - Temporal trends in age-adjusted mortality by dementia in Cuba between 2000 and 2019


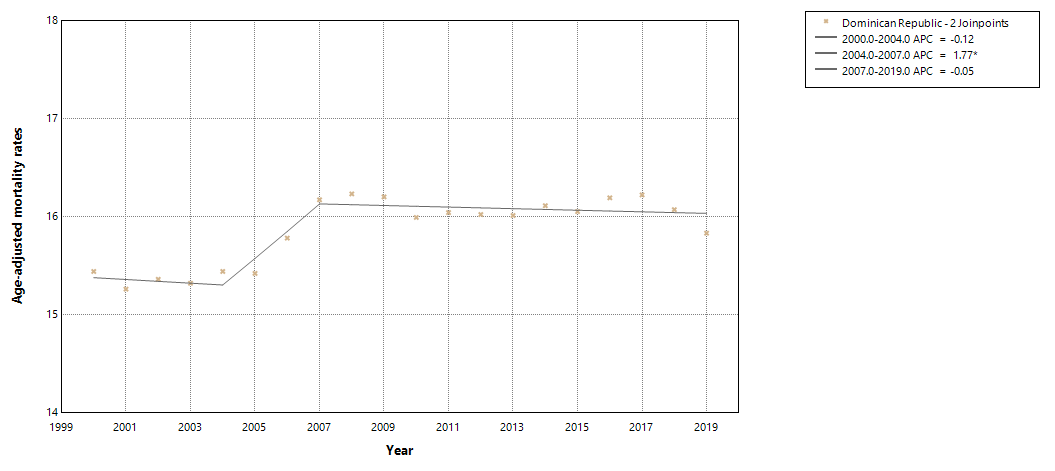


Supplementary figure 13 - Temporal trends in age-adjusted mortality by dementia in Dominican Republic between 2000 and 2019


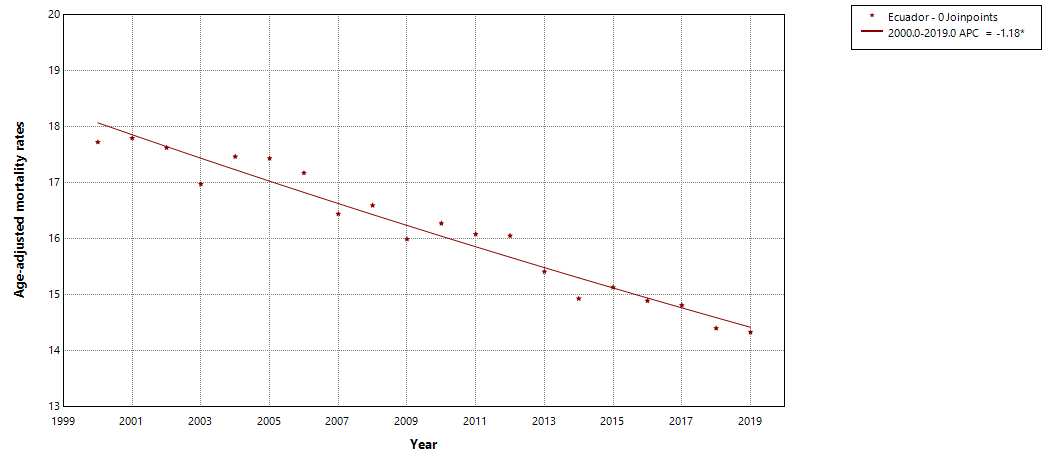


Supplementary figure 14 - Temporal trends in age-adjusted mortality by dementia in Ecuador between 2000 and 2019


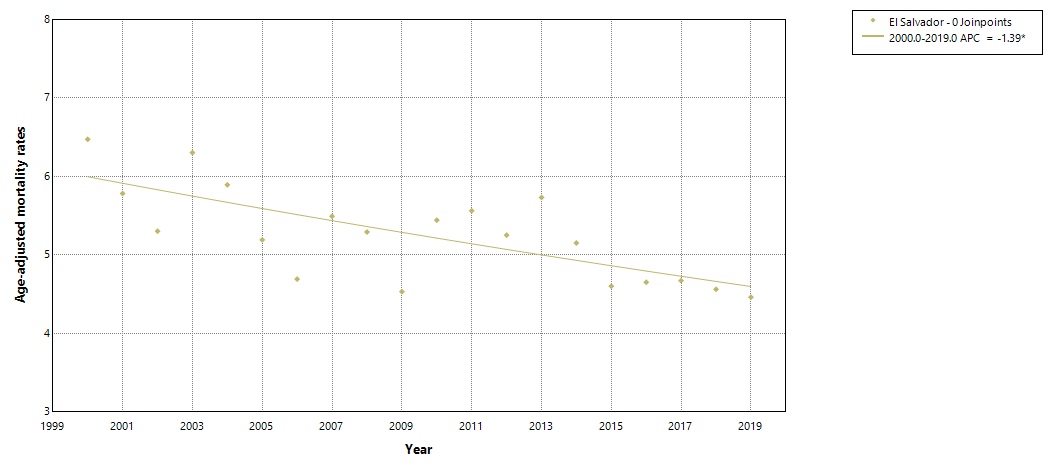


Supplementary figure 15 - Temporal trends in age-adjusted mortality by dementia in El Salvador between 2000 and 2019


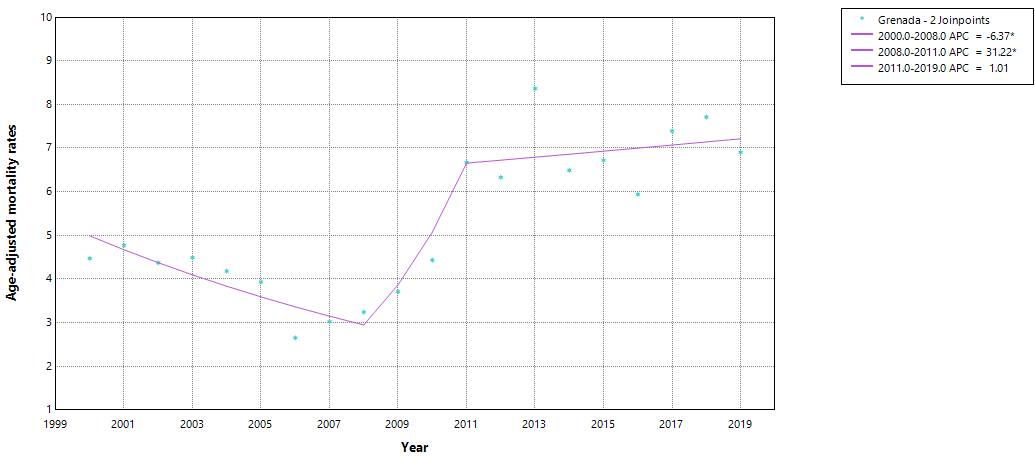


Supplementary figure 16 - Temporal trends in age-adjusted mortality by dementia in Grenada between 2000 and 2019


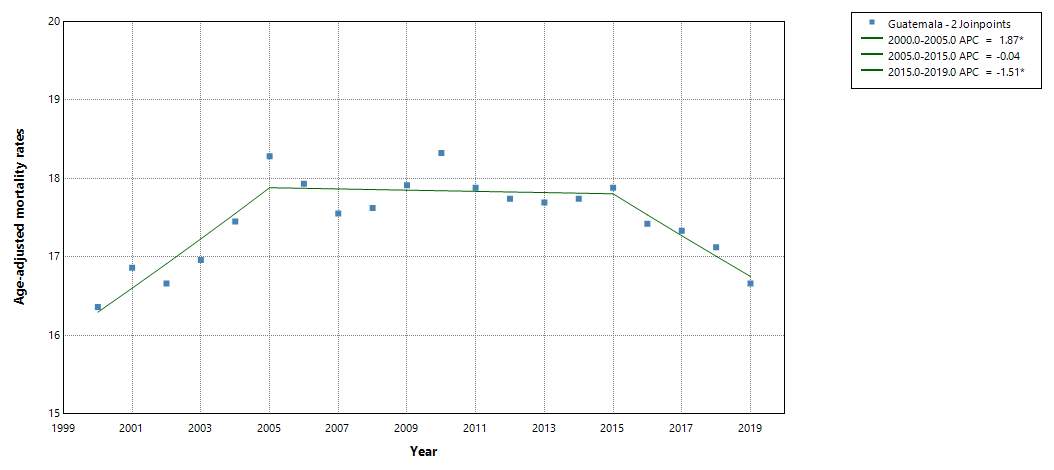


Supplementary figure 17 - Temporal trends in age-adjusted mortality by dementia in Guatemala between 2000 and 2019


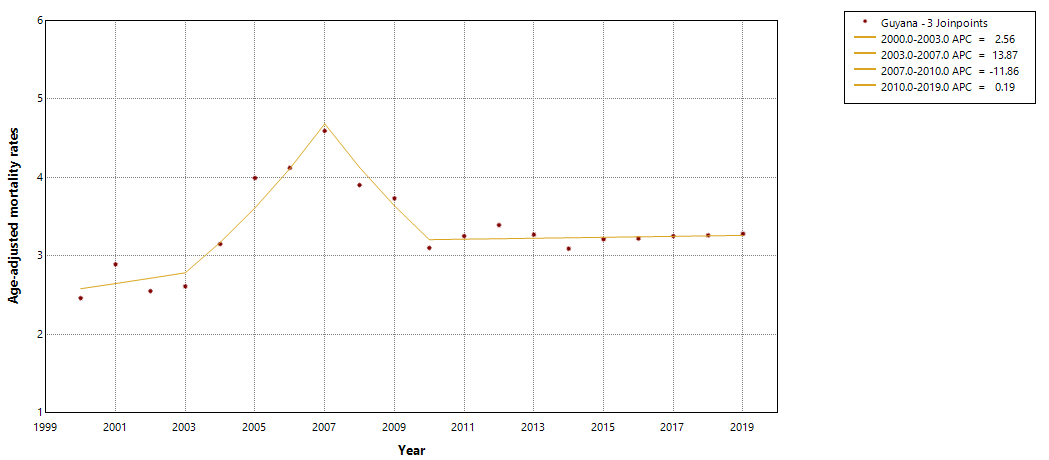


Supplementary figure 18 - Temporal trends in age-adjusted mortality by dementia in Guyana between 2000 and 2019


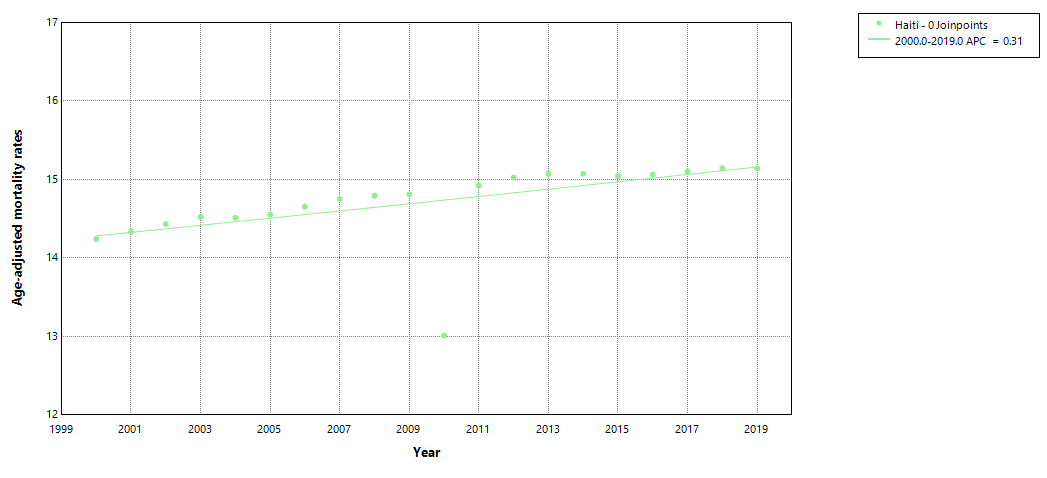


Supplementary figure 19 - Temporal trends in age-adjusted mortality by dementia in Haiti between 2000 and 2019


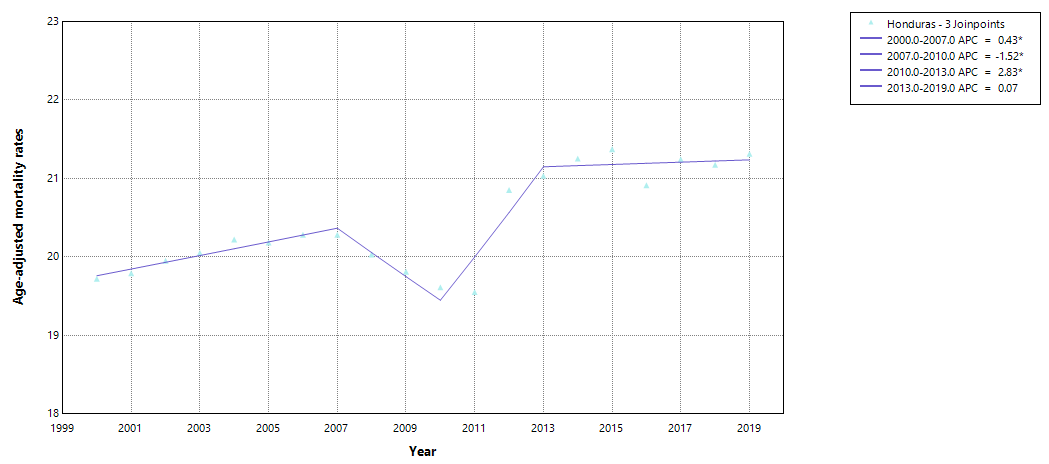


Supplementary figure 20 - Temporal trends in age-adjusted mortality by dementia in Honduras between 2000 and 2019


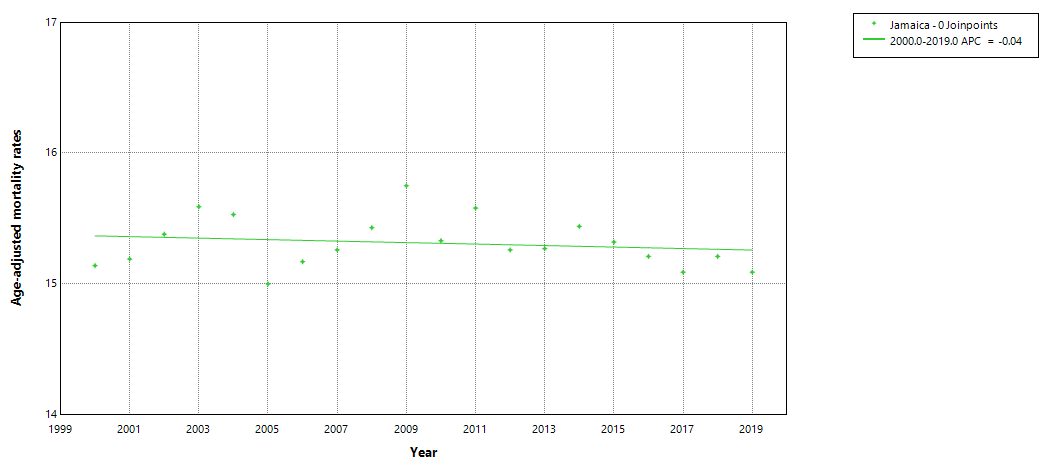


Supplementary figure 21 - Temporal trends in age-adjusted mortality by dementia in Jamaica between 2000 and 2019


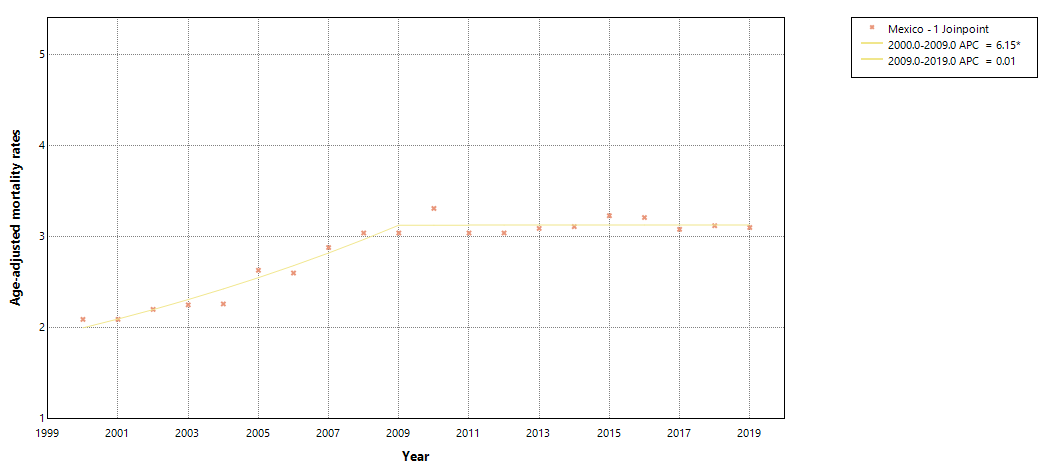


Supplementary figure 22 - Temporal trends in age-adjusted mortality by dementia in Mexico between 2000 and 2019


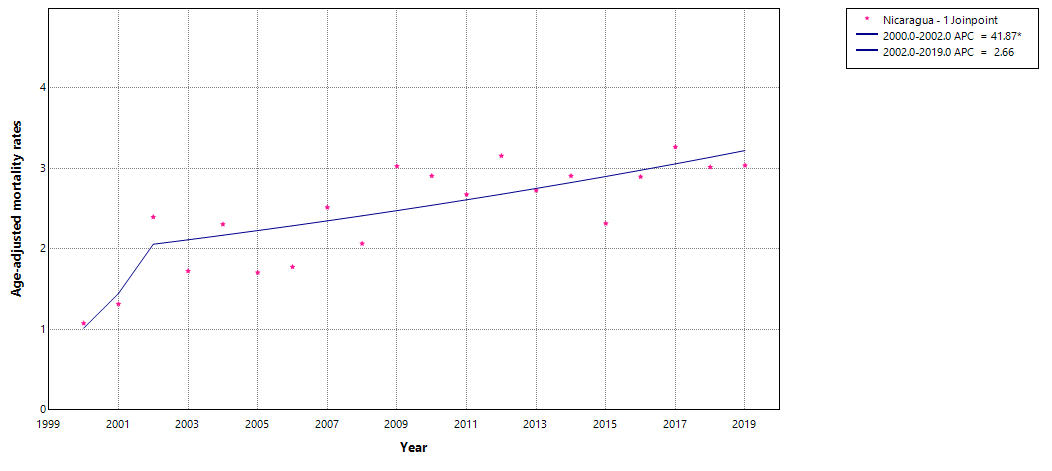


Supplementary figure 23 - Temporal trends in age-adjusted mortality by dementia in Nicaragua between 2000 and 2019


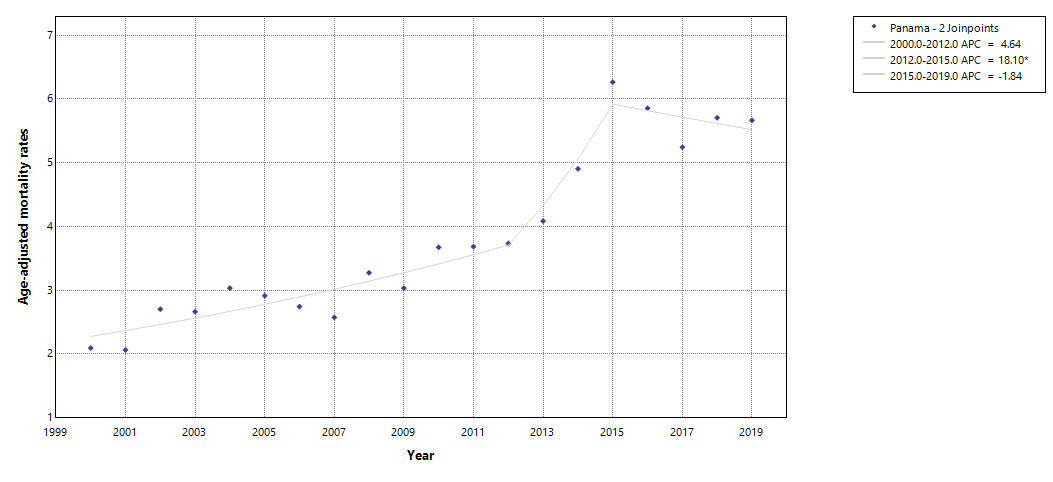


Supplementary figure 24 - Temporal trends in age-adjusted mortality by dementia in Panama between 2000 and 2019


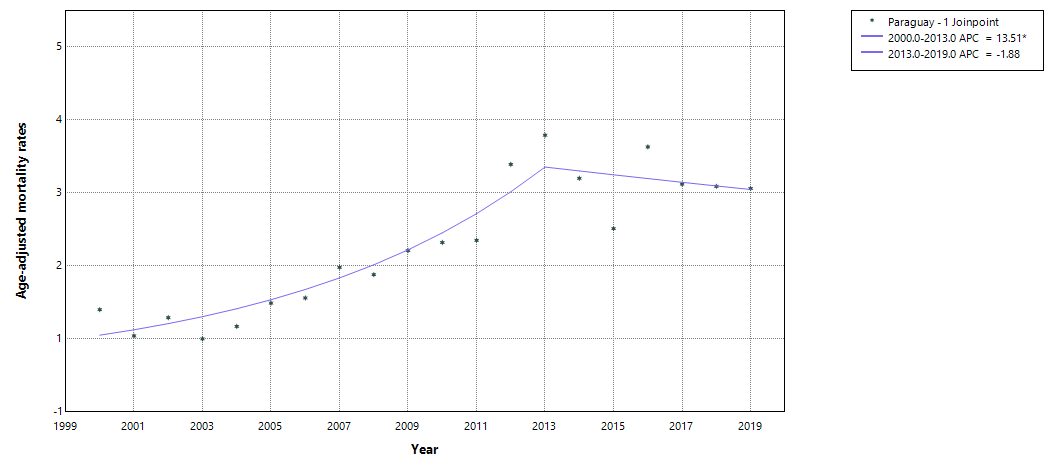


Supplementary figure 25 - Temporal trends in age-adjusted mortality by dementia in Paraguay between 2000 and 2019


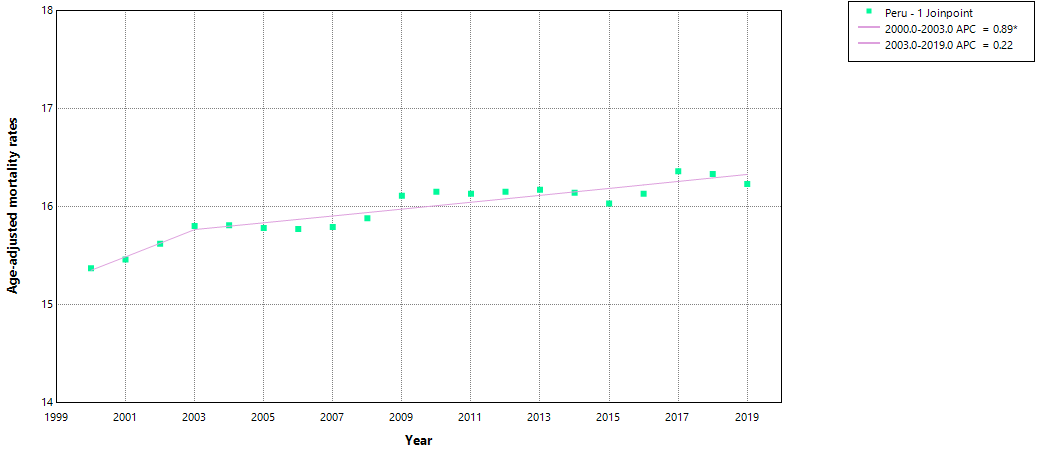


Supplementary figure 26 - Temporal trends in age-adjusted mortality by dementia in Peru between 2000 and 2019


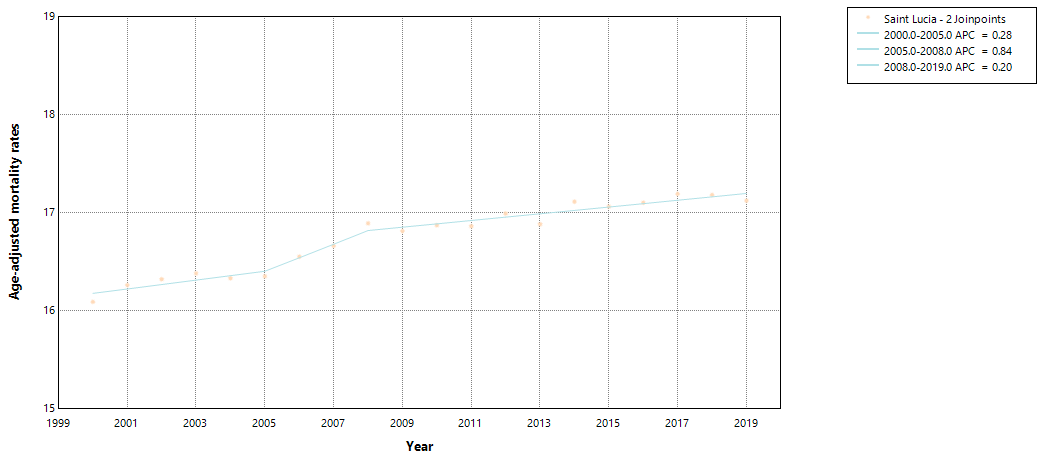


Supplementary figure 27 - Temporal trends in age-adjusted mortality by dementia in Saint Lucia between 2000 and 2019


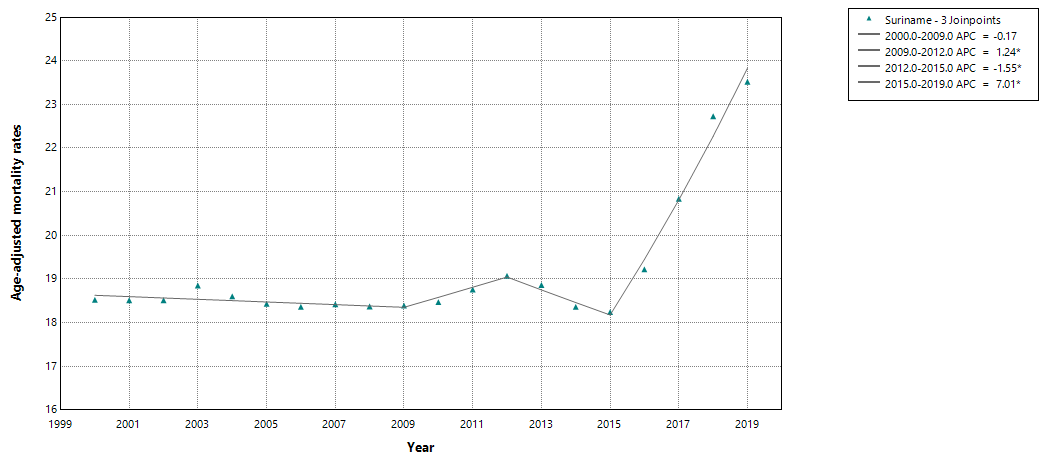


Supplementary figure 28 - Temporal trends in age-adjusted mortality by dementia in Suriname between 2000 and 2019


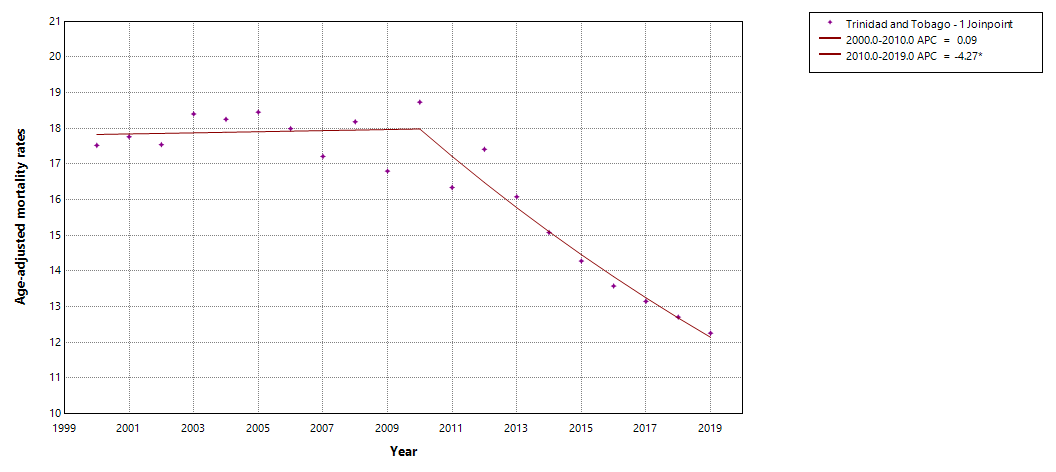


Supplementary figure 29 - Temporal trends in age-adjusted mortality by dementia in Trinidad e Tobago between 2000 and 2019


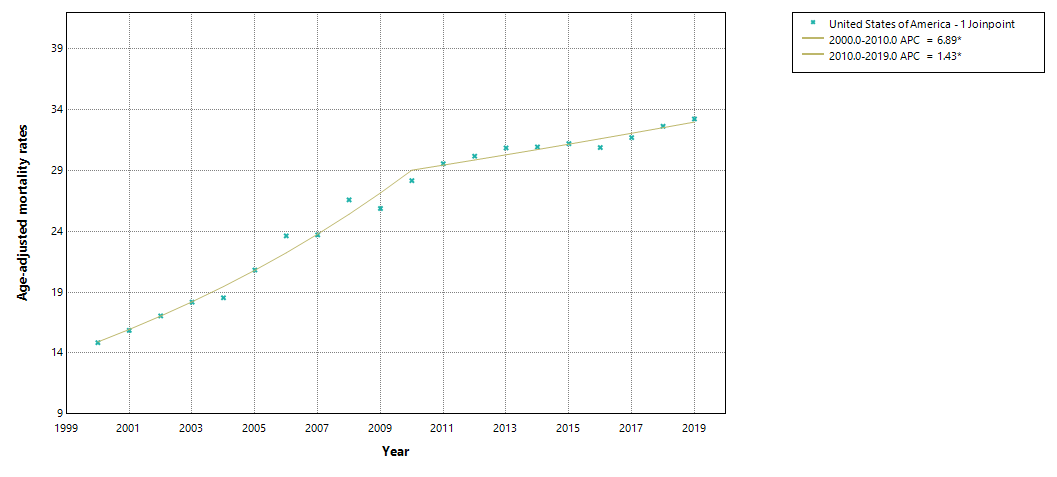


Supplementary figure 30 - Temporal trends in age-adjusted mortality by dementia in United States of America between 2000 and 2019


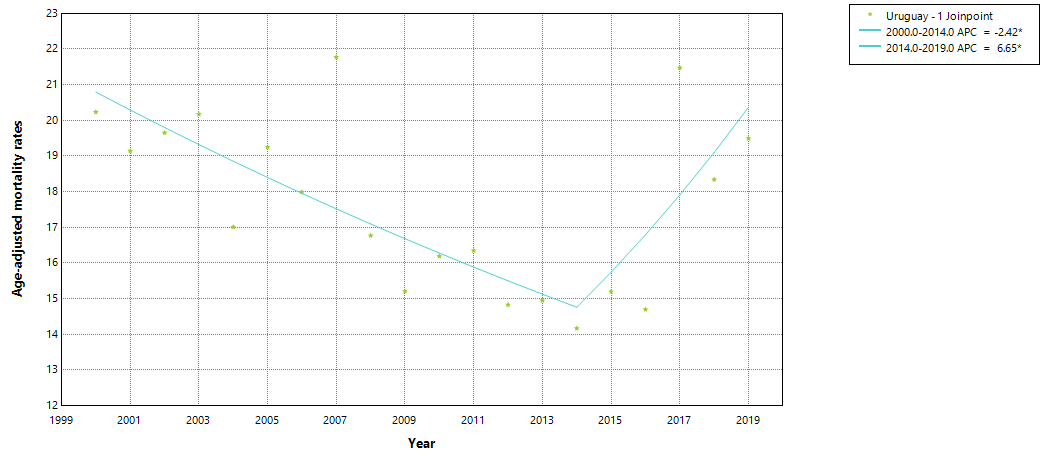


Supplementary figure 31 - Temporal trends in age-adjusted mortality by dementia in Uruguay between 2000 and 2019


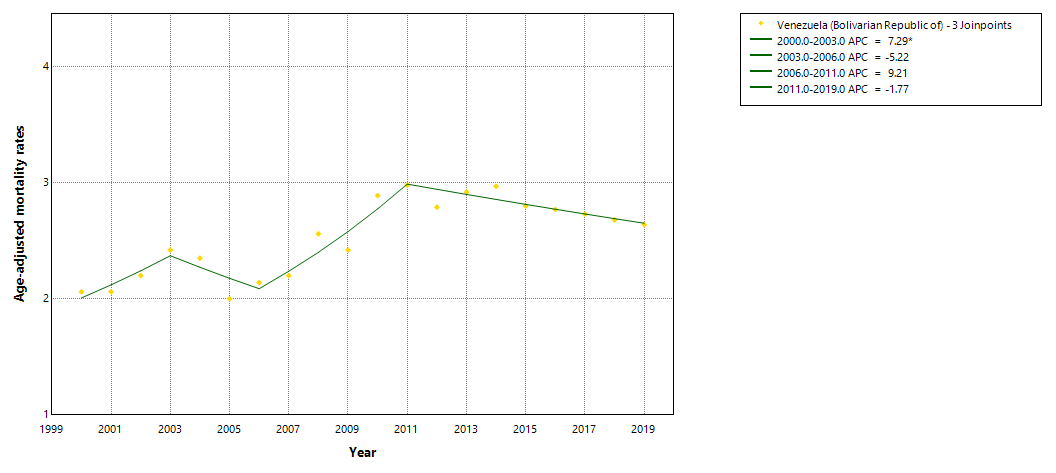


Supplementary figure 32 - Temporal trends in age-adjusted mortality by dementia in Venezuela between 2000 and 2019


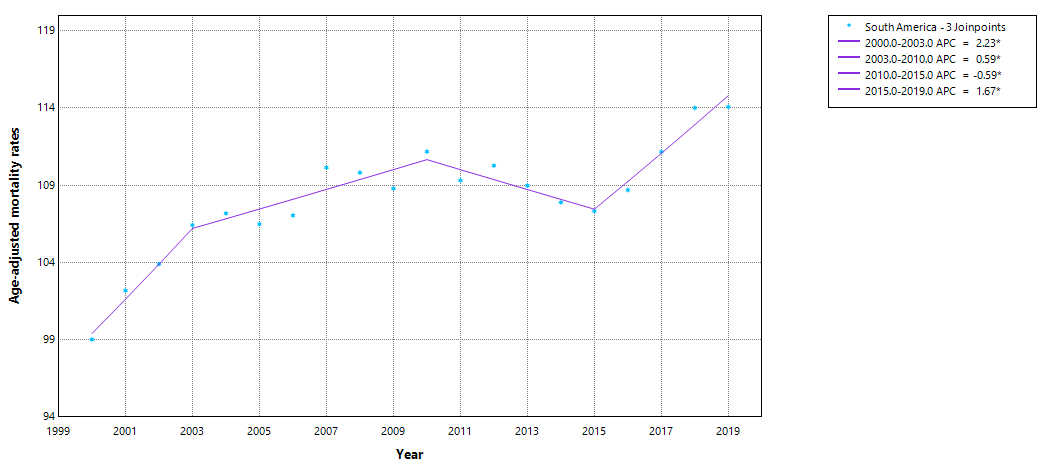


Supplementary figure 33 - Temporal trends in age-adjusted mortality by dementia in South America between 2000 and 2019


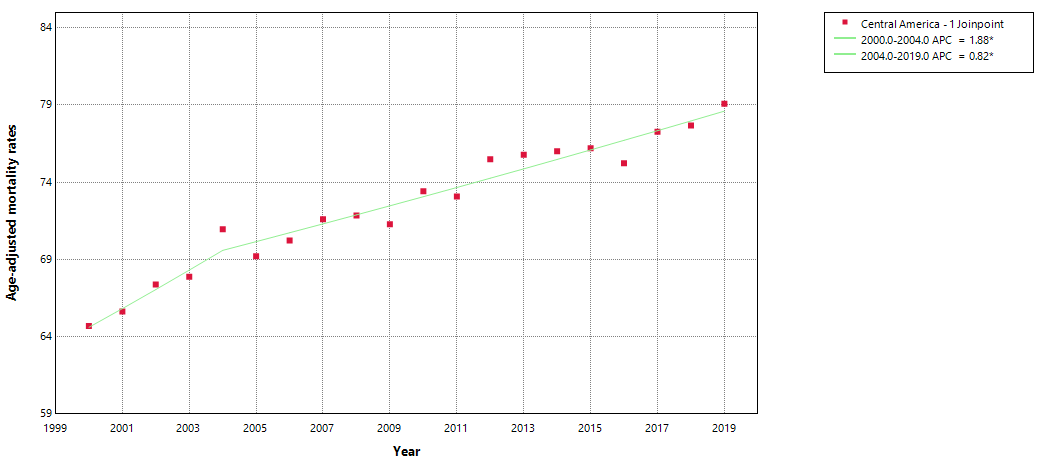


Supplementary figure 34 - Temporal trends in age-adjusted mortality by dementia in Central continental America between 2000 and 2019


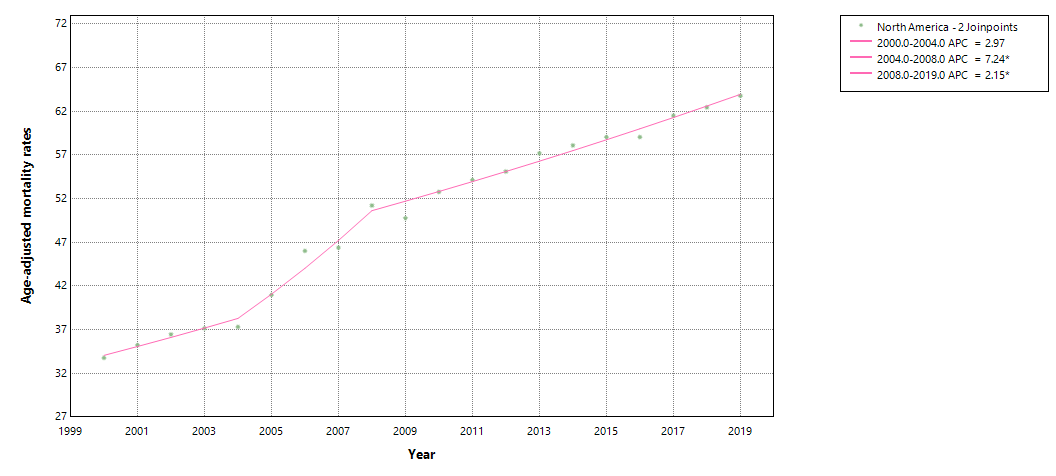


Supplementary figure 35 - Temporal trends in age-adjusted mortality by dementia in North America between 2000 and 2019


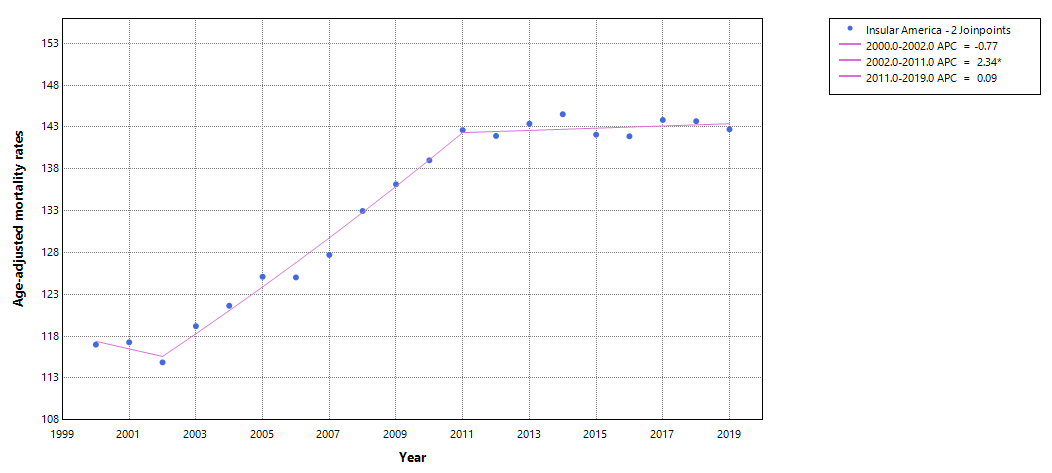


Supplementary figure 36 - Temporal trends in age-adjusted mortality by dementia in Insular America between 2000 and 2019
